# Psychosocial factors affect the occurrence of nonsuicidal self-injury in adolescents with major depressive disorder through chain mediation

**DOI:** 10.1007/s00406-024-01858-0

**Published:** 2024-07-08

**Authors:** Tian Ren, Yujiao Wen, Lu Ma, Dan Qiao, Gaizhi Li, Hong Li, Xiao Wang, Zhifen Liu

**Affiliations:** 1 Department of Psychiatry, First Hospital of Shanxi Medical University, NO. 85 Jiefang South Road, Taiyuan, China; 2 Shanxi Key Laboratory of Artificial Intelligence Assisted Diagnosis and Treatment for Mental Disorder, First Hospital of Shanxi Medical University, Taiyuan, China

**Keywords:** Major depressive disorder, Nonsuicidal self-injury, Adolescent, Psychosocial factors

## Abstract

In the adolescent group, about half of adolescents with major depressive disorder (MDD) have NSSI. Psychosocial factors are associated with the development of NSSI. Clarifying the relationship between psychosocial factors and NSSI in adolescents with MDD can help us achieve early prevent. Demographic data, Hamilton Depression Scale-24 (HAMA_24_), childhood trauma questionnaire, emotional intelligence scale and interpersonal reactivity index were collected from 187 adolescents with MDD. Use ANOVA, Chi-square test, Binary Logistic Regression, Pearson correlation analysis, Mediation effect analysis and the Structural Equation Model for data analysis. The results of ANOVA showed that there was significant difference between the two groups in HAMD_24_ total score, impulsiveness, emotional intelligence, and empathy (*p* < 0.05). In the regression analysis, women, depression degree, motor impulsiveness (MI), personal distress (PD) and appraisal of other’s emotions empathy were the risk factors for MDD adolescents to produce NSSI behavior. Among the indicators that were significantly related to MDD and NSSI, MI and PD mediate the relationship between MDD and NSSI. The structural equation model showed that MDD, PD and MI had a direct impact on NSSI, but PD and MI had multiple intermediary effected in the relationship between MDD and NSSI. Emotional intelligence, emotional neglect and cognitive impulsiveness indirectly affected the occurrence of NSSI behavior. Impulsiveness, personal distress, emotional neglect, and emotional intelligence are important risk factors that affect NSSI behavior in adolescents with MDD, and they affect the occurrence of NSSI in adolescents with MDD through chain mediation.

## Introduction

Major depressive disorder (MDD) was a type of mental disorder caused by a variety of reasons, and accompanied by varying degrees of cognitive and behavioral changes, such as some patients have self-injurious behavior and SI, severe conditions may lead to death [1]. The World Health Organization (WHO) has ranked MDD as the second leading cause of disability globally and is the leading burden of disease globally among adolescents [2]. Adolescence is a period of significant increase in the incidence of depressive disorder [3]. After adolescents suffer from negative stimuli, they are more likely to produce negative emotions and (or) impulsive behaviors, such as NSSI.

Non-suicidal self-injury (NSSI) refers to repetitive, purposeful acts that harm one’s own body tissues without the purpose of suicide, such as scratching or scalding the skin, hitting oneself [4]. The age of onset is 12–15 years old, and the prevalence reached its peak at 15–16 years old, and begins to weaken in adulthood [5, 6]. Studies in recent years have found that people with depression are prone to NSSI, more than one time was reported in 37% of depressed patients [7], and the proportion of NSSI in adolescents with mental disorders was as high as 40-87% [8]. Adolescents who practiced NSSI behaviors had a significantly higher risk of suicidal thoughts and suicidal behaviors in the next year, 1.6–4.1 times higher than adolescents without NSSI behaviors [9]. Therefore, early identification and intervention of adolescents with depression and individuals with NSSI is necessary.

Persistent negative emotional states, is an essential factor in the persistence of NSSI behavior [10]. In a study of 444 MDD undergraduates, after controlling gender and age, it was found that childhood abuse affected the occurrence of NSSI behavior in MDD patients [11]. In the study of bipolar depression adolescents, it was found that only emotional abuse affected the occurrence of NSSI behavior [12]. Therefore, in the integrated model, childhood abuse is one of the remote risk factors that induce NSSI, at the same time, which may lead to abnormal emotional regulation and interpersonal communication in the later life of individuals [13]. Due to the lack of effective emotional regulation strategies, individuals are more likely to choose NSSI as a quick and effective way to relieve emotions after experiencing negative emotions [14].When adolescents adopt a friendly, open, empathy and non-evasive way to face negative situations and reduce the negative impact [15], that is, adaptive emotional regulation can protect against the individual’s participation in the psychopathy-related schema after suffering from adverse events [16]. In addition to this, an individual’s personality traits may be related to the production of NSSI behavior. In some, NSSI is a mechanism to release emotional tension, and in others a method, albeit maladaptive, to self-regulate volatile emotions [17]. When experiencing the same degree of negative events, teenagers with high impulse may choose NSSI as the way of emotional regulation [18].

To sum up, a significant number of NSSI are random impulsive acts in the context of psychological distress and vulnerability or actual psychopathology. Previous studies exploring the influencing factors of NSSI behavior in MDD patients have been limited, with most studies using depressive symptoms or severity as mediating factors to explore the relationship between a certain factor and NSSI, such as childhood abuse [19], impulsiveness level [20], emotional regulation [21] and empathy [22] may affect NSSI behavior. However, at present, it is not clear how these psychosocial factors differ between MDD adolescents with and without NSSI, and how these factors interact with each other, and to what extent they can affect the occurrence of NSSI behavior.

The purpose of this study is to: (1) explore whether there are differences in psychosocial factors between adolescents with and without NSSI behavior in MDD; (2) determine which psychosocial factors will affect the occurrence of NSSI behavior in MDD adolescents and whether these factors interact. Because of the high prevalence of MDD with NSSI in adolescents, adolescents were selected as the study subjects.

## Methods

### Participants

Participants were primarily from outpatients and inpatients of mental health department of the First Hospital of Shanxi Medical University. The recruitment lasted one and a half years from March 2021 to September 2022, and a total of 187 adolescents were referred for participation. This study was approved by the Ethics Committee of the First Hospital of Shanxi Medical University and written informed consent was obtained from all the participants and from the legal guardians of the participants who were below 16 years of age after a thorough description of the study, including details about potential risks, benefits and reporting of harms, had been provided. When the informed consent was signed, two intake worker conducted an initial assessment with adolescent or a parent.

### Inclusion and exclusion criteria

Inclusion criteria of patients were as follows: (1) age 12 through 23 years [23]; (2) met the Diagnostic and Statistical Manual of Mental Disorders Fifth Edition (DSM-V) criteria for MDD as assessed on the SCID-5-RV; (3) not taking medication or taking medication regularly prior to baseline assessment; (4) voluntarily participated in the trial and signed an informed consent form. If participant was under 18 years old, the guardian would sign the informed consent form with them. When a subject met one of the following criteria, the subject cannot participate in the trail, such as reported a previous manic or hypomanic episode, had a bipolar disorder, schizophrenia, or other disorders related to mental disorders on the SCID-5-RV or reported alcohol, psychoactive substance dependence/abuse, had a history of neurological disease (e.g. brain trauma, epilepsy, etc.) or structural abnormalities in the brain shown on MRI or physical illness impairing their ability to participate in a group program.

### Measures

A screen for inclusion and exclusion criteria, and the SCID-5-RV Mood Episodes Module from A to D. Those administering the SCID-5-RV were trained psychiatrists who had all completed the standard SCID training.

The assessment included the gathering of family socioeconomic and demographic information ( i.e.,age, gender, education, studying status, chief complaint and present medical history in the adolescents ).

*Ottawa Self-injury Inventory* [24] was primarily used to measure non-suicidal self-injury in adolescents, and the frequency of NSSI among adolescents in the study was analysed using data from item 1 ”In the past 1 year and in the past 1 month, self-injurious behavior has been committed, but it is not suicidal”.

*Hamilton Depression Scale-24* (HAMD_24_) was a 24-item scale conducted by psychiatrists to measure a patient’s severity of depression, and most of its item use a 5-scale scale of 0 to 4. A total score greater than 8 is considered to be depressive.

*Childhood Trauma Questionnaire* [25] was used to investigate the growth experience in childhood (before the age of 16) and included five subscales of physical abuse, emotional abuse, sexual abuse, physical neglect and emotional neglect. The scale used a 5-level score, and the higher the score, the more abuse.

*Barratt Impulsiveness Scale 11th version* [26] The revised Chinese Barratt impulsivity personality scale (BIS-11) was used to measure impulsivity, which consisted of 30 items and included three dimensions: motor impulsiveness, cognitive impulsiveness, and no planning impulsiveness. The higher the score, the more impulsive it is.

*Emotional intelligence scale* [27] was used to assess individual’s ability to perceive, understand, express, control, and manage their own and other’s emotions. It was divided into 4 dimensions: appraisal of own emotions, regulation of own emotions, appraisal of other’s emotions empathy, and utilization of emotions. A higher score means that individual can express feelings more clearly, had better response and had fewer emotional obstacles. In this study, emotional regulation will be included in the measurement of emotional intelligence as a dimension.

*Interpersonal Reactivity Index* [28] was used to measure the empathy ability of individuals. It was divided into 4 subscales: perspective taking (individual’s tendency to adopt others’ opinions spontaneously); fantasy scale (empathize with fictional characters); empthetic concern (individual concern and sympathy for people in distress); and personal distress (feeling anxious and uncomfortable in interpersonal situations). It adopted a 5-level scoring system. The higher the score, the better the ability.

### Statistical analysis

#### Comparing differences in psychosocial factors

The data were analyzed using SPSS Statistics version 26.0. Chi-square test was used for categorical variables in general demographic data, such as gender. T test or analysis of variance were used for continuous variables. Means and standard deviations or median and inter-quartile ranges were computed for normally and non-normally distributed variables. Categorical variables were expressed as proportions. Level of significance was set at 5%.

#### Psychosocial factors’ regression equation and correlation

Binary logistic regression and Pearson correlation analysis were used to detect correlations between depression severity and psychosocial factors measured using the above scales. In the regression equation, factors with an odds ratios (OR) of more than 1 are risk factors. The continuous variables were centralized, the mediation model is constructed for factors related to both MDD and NSSI. The PROCESS v3.5 plugin was used for mediation analysis of the data.

#### Building the structural equation model

According to the factors with differences in the analysis of variance and the risk factors shown in the logistic regression, using AMOS software to construct and test the structural equation model (SEM). In SEM, several criteria, such as, root mean square error of approximation (RMSEA) values lower than 0.08, and comparative fit index (CFI) and Tucker Lewis index (TLI) values greater than 0.90, indicated better models.

## Results

At the beginning of the experiment, a total of 196 adolescents were recommended to take part in the study, which was initially checked for compliance with the inclusion criteria. Of these, 9 adolescents were unable to participate in the study due to time, not meeting the inclusion criteria or other reasons.

### Demographic and baseline characteristics

**Fig. 1:**
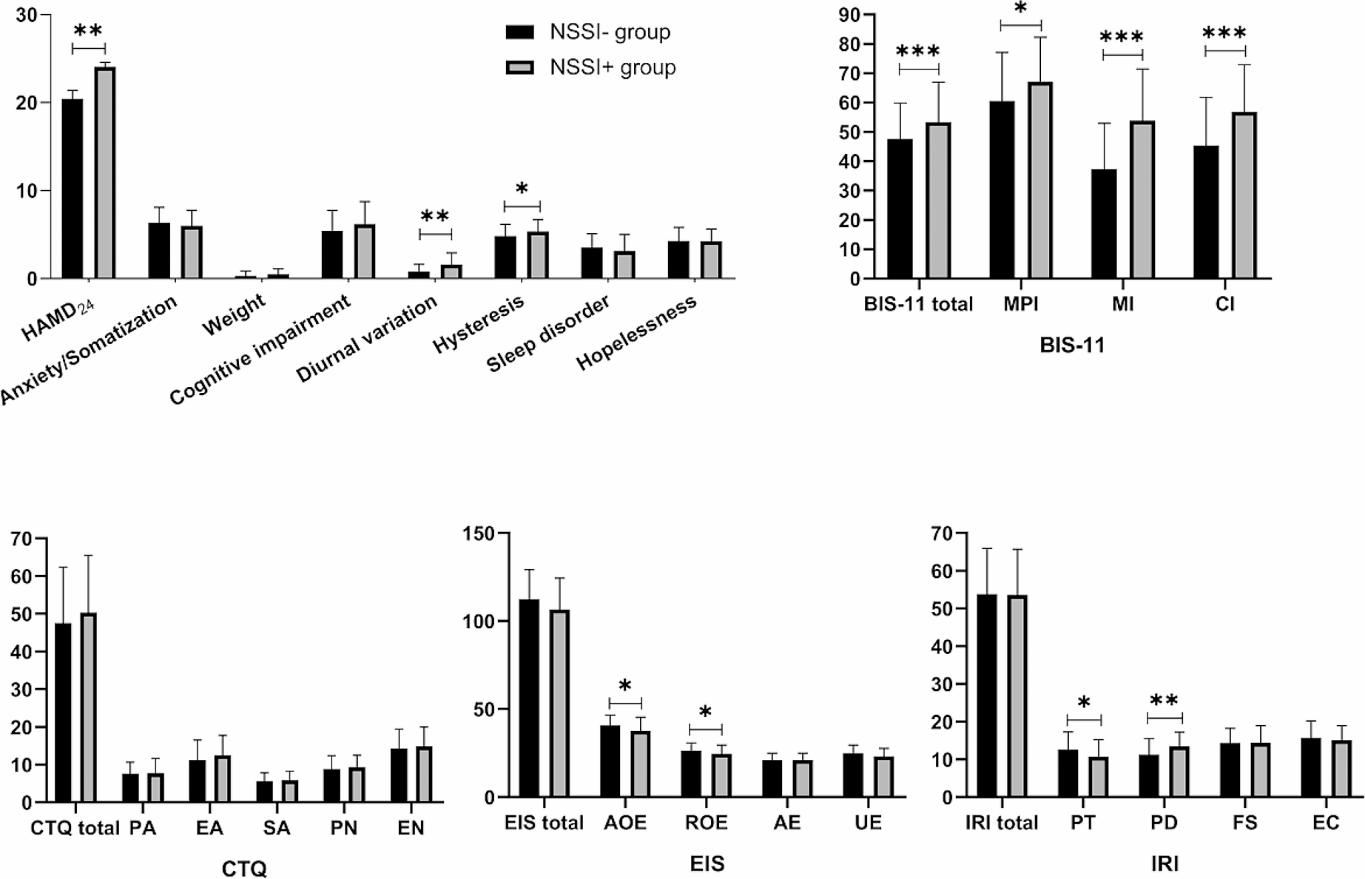
Difference in psychosocial factors between MDD patients with and without NSSI. NSSI-= major depressive disorder without nonsuicidal self-injury; NSSI + = major depressive disorder with nonsuicidal self-injury; HAMD_24_ = Hamilton depression scale-24; CTQ = Childhood trauma questionnaire; EIS = Emotional intelligence scale; IRI = Interpersonal reactivity index; AOE = Appraisal of own emotions; ROE = Regulation of own emotions; AE = Appraisal of other’s emotions empathy; UE = utilization of emotions. PA = Physical abuse; EA = Emotional abuse; SA = sexual abuse; PN = physical neglect; EN = Emotional neglect; NPI = No planning impulsiveness; MI = Motor impulsiveness; CI = Cognitive impulsiveness; PT = Perspective taking; PD = Personal distress; FS = Fantasy scale; EC = Empthetic concern.*: *P* < 0.05; **: *P* < 0.01;***: *P* < 0.001

Chi square and independent sample test were run to compare demographics across NSSI- group (*n* = 84) and NSSI + group (*n* = 103). 55.1% of adolescents had a history of NSSI. In the NSSI + group, one month before the survey, the number of NSSI behaviors performed by adolescent was 3.84; The number of times in the past year was 12.89. There was significant difference with the NSSI- groups (Table 1). In addition, there were significant differences between the two groups in the gender (*p* = 0.012), age (*p* = 0.007), severity of depression (*p* = 0.001), diurnal variation (*p* = 0.001), hysteresis (*p* = 0.035), various dimensions of impulsiveness (BIS-11) (*p* < 0.001), AOE (*p* = 0.026), ROE (*p* = 0.034), perspective taking (*p* = 0.028) and personal distress dimensions (*p* = 0.002) (see Fig. 1).

**Table 1:** Difference in demographic characteristics and psychosocial factors between MDD patients with and without NSSI.

Dimension		NSSI- ( *n* = 84)	NSSI+ ( *n* = 103)	*P*-value	
Gender,					
Female		51	80	0.012*	
Male		33	33		
Age (M ± SD)		17.32 ± 2.41	15.99 ± 2.57	0.007**	
Education				0.228	
≤ 9 years		22	40		
9 ~ 12 years		47	47		
> 12 years		15	16		
HAMD_24_ Total (M ± SD)		20.43 ± 5.47	24.06 ± 6.03	0.001**	
	Anxiety/Somatization	6.33 ± 1.77	6.00 ± 1.75	0.312	
	Weight	0.31 ± 0.52	0.48 ± 0.64	0.144	
	Cognitive impairment	5.44 ± 2.30	6.18 ± 2.55	0.114	
	Diurnal variation	0.81 ± 0.79	1.60 ± 1.31	0.001**	
	Hysteresis	4.83 ± 1.32	5.36 ± 1.35	0.035*	
	Sleep disorder	3.53 ± 1.56	3.14 ± 1.87	0.256	
	Hopelessness	4.28 ± 1.52	4.26 ± 1.35	0.945	
CTQ Total (M ± SD)		47.54 ± 14.79	50.33 ± 15.21	0.322	
	Physical abuse	7.60 ± 3.06	7.75 ± 3.89	0.823	
	Emotional abuse	11.24 ± 5.32	12.53 ± 5.22	0.184	
	Sexual abuse	5.60 ± 2.22	5.88 ± 2.40	0.530	
	Physical neglect	8.85 ± 3.53	9.31 ± 3.20	0.447	
	Emotional neglect	14.26 ± 5.17	14.86 ± 5.10	0.530	
BIS−11 Total (M ± SD)		47.57 ± 12.26	58.38 ± 13.55	< 0.001***	
	No planning impulsiveness	60.55 ± 16.54	67.06 ± 15.15	0.024*	
	Motor impulsiveness	37.34 ± 15.56	53.87 ± 17.55	< 0.001***	
	Cognitive impulsiveness	45.41 ± 16.37	56.88 ± 15.99	< 0.001***	
EIS Total (M ± SD)		112.44 ± 16.75	106.50 ± 17.94	0.072	
	AOE	40.72 ± 5.79	37.63 ± 7.77	0.026*	
	ROE	26.49 ± 4.35	24.54 ± 5.03	0.034*	
	AE	20.92 ± 4.00	21.04 ± 3.84	0.869	
	UE	24.78 ± 4.79	23.18 ± 4.51	0.061	
IRI Total (M ± SD)		53.84 ± 12.09	53.58 ± 12.12	0.908	
	Perspective taking	12.59 ± 4.73	10.72 ± 4.52	0.028*	
	Personal distress	11.22 ± 4.24	13.45 ± 3.78	0.002**	
	Fantasy scale	14.33 ± 3.89	14.36 ± 4.65	0.964	
	Empthetic concern	15.70 ± 4.54	15.05 ± 3.85	0.380	

MDD = major depressive disorder without nonsuicidal self-injury behavior; NSSI + = nonsuicidal self-injury ; HAMD_24_ = Hamilton depression scale-24; CTQ = Childhood trauma questionnaire; EIS = Emotional intelligence scale; IRI = Interpersonal reactivity index; AOE = Appraisal of own emotions; ROE = Regulation of own emotions; AE = Appraisal of other’s emotions empathy; UE = utilization of emotions

*: *P* < 0.05; **: *P* < 0.01; ***: *P* < 0.001

### Binary logistic regression

**Fig. 2:**
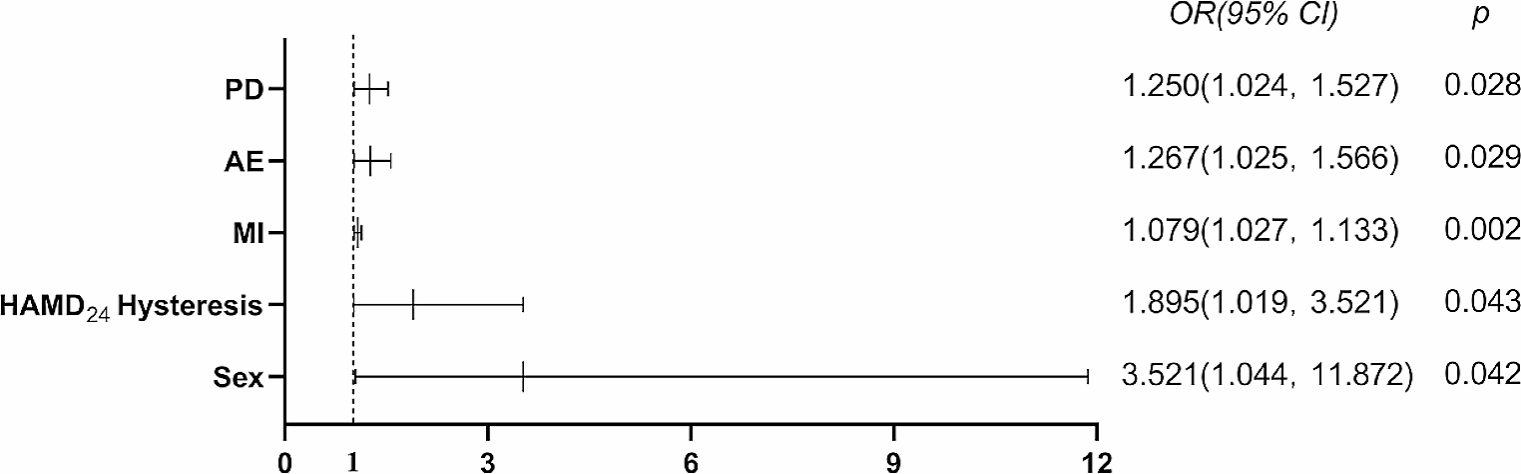
Risk factors among multiple variables in binary logistic regression. *: *P* < 0.05; **: *P* < 0.0.

In order to better understand how the above psychosocial factors cause danger or protection to NSSI behavior, a binary logistic regression analysis was carried out and the results were shown in Fig. 2. All psychosocial dimensions in this study were included in the equation, and statistics of the Hosmer-Lemeshow test reached the maximum value of 0.733. The significance was greater than 0.05, indicating that the equation fitting effect was ideal (Table 2).

**Table 2:** Relationships among multiple variables in binary logistic regression

Dimension	Estimate	SE	*P*-value	OR (95% CI)	
age	−0.213	0.141	0.132	0.808(0.612,1.066)	
sex	1.259	0.620	0.042*	3.521(1.044,11.872)	
Anxiety/Somatization	−0.181	0.189	0.338	0.834(0.576,1.209)	
Weight	1.554	0.660	0.109	4.731(1.297,17.262)	
Cognitive impairment	0.046	0.161	0.776	1.047(0.764,1.435)	
Diurnal variation	0.582	0.308	0.059	1.789(0.977,3.275)	
Hysteresis	0.639	0.316	0.043*	1.895(1.019,3.521)	
Sleep disorder	−0.459	0.20	0.064	0.632(0.424,0.942)	
Hopelessness	−0.059	0.285	0.837	0.943(0.540,1.648)	
Physical abuse	−0.073	0.119	0.541	0.930(0.736,1.175)	
Emotional abuse	−0.063	0.100	0.528	0.939(0.771,1.142)	
Sexual abuse	0.099	0.163	0.544	1.104(0.802,1.520)	
Physical neglect	−0.012	0.144	0.934	0.988(0.745,1.310)	
Emotional neglect	0.062	0.087	0.475	1.064(0.897,1.263)	
No planning impulsiveness	−0.030	0.030	0.310	0.970(0.915.1.029)	
Motor impulsiveness	0.076	0.025	0.002**	1.079(1.027,1.133)	
Cognitive impulsiveness	−0.013	0.035	0.701	0.987(0.921,1.057)	
AOE	−0.059	0.060	0.321	0.943(0.839,1.059)	
ROE	−0.230	0.127	0.071	0.795(0.619,1.020)	
AE	0.237	0.108	0.029*	1.267(1.025,1.566)	
UE	0.047	0.131	0.720	1.048(0.811,1.354)	
Perspective taking	−0.138	0.088	0.118	0.871(0.733,1.035)	
Personal distress	0.223	0.102	0.028*	1.250(1.024,1.527)	
Fantasy scale	−0.034	0.111	0.762	0.967(0.778,1.202)	
Empthetic concern	−0.022	0.087	0.797	0.978(0.824,1.160)	
Constant	−0.177	4.3	0.803	0.838	

*: *P* < 0.05; **: *P* < 0.01; ***: *P* < 0.001

In addition to female adolescents were more likely to have NSSI behaviors than men, with a risk of 3.521 times that of men. Hysteresis, motor impulsiveness, appraisal of other’s emotions empathy and personal distress were risk factors affecting NSSI behavior. The score of the equivalent table increased by 1 unit, and the risk of NSSI behavior increased by 89.5%, 7.9%, 26.7% and 25.0% in each factor.

### Correlation between MDD, NSSI and psychosocial factors

**Fig. 3:**
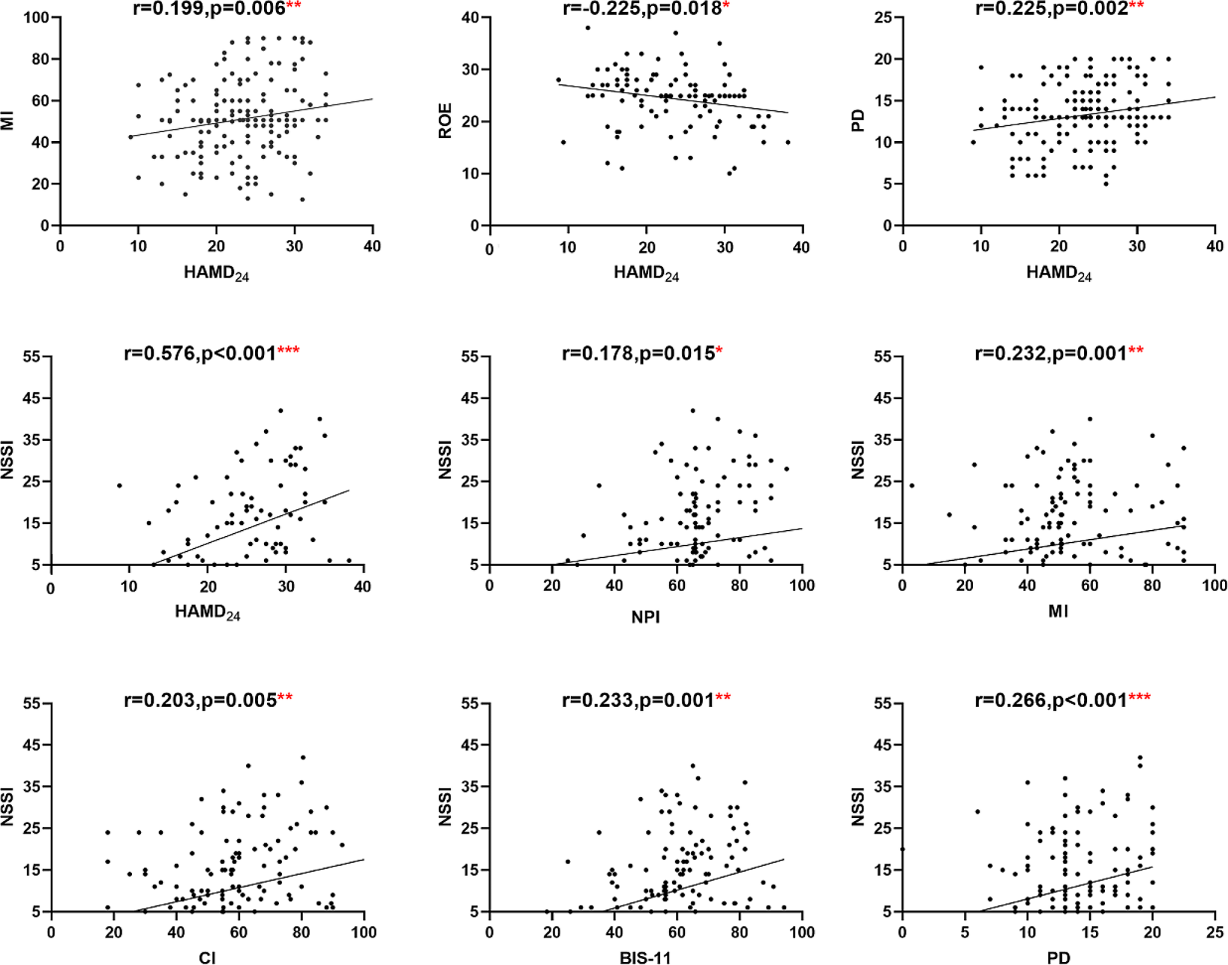
Pearson correlation matrix of MDD, NSSI and psychosocial factors. *: *P* < 0.05; **: *P* < 0.01; ***: *P* < 0.001

The HAMD_24_ and NSSI would be correlated with the following psychosocial factors: Total score and each subscale of BIS-11, appraisal of own emotions, regulation of own emotions, appraisal of other’s emotions empathy, perspective taking, personal distress, and childhood abuse, which was considered as risk factors in previous studies. Figure 3 showed the correlations between MDD, NSSI and other psychosocial factors. Significant positive correlation was observed between MDD and NSSI. No planning impulsiveness, cognitive impulsiveness and BIS-11 were significantly positively correlated with NSSI. MDD was significantly negatively with regulation of own emotions. Motor impulsiveness and personal distress were significantly correlated with MDD and NSSI, which was consistent with the premise of mediation (Tables 3 and 4).

**Table 3:** Pearson correlation matrix of MDD with NSSI and psychosocial factors

	NSSI		MDD		
	*r*	*P*-value		*r*	*P*-value
MDD	0.576	< 0.001***		-	-
PA	0.080	0.278		−0.105	0.154
EA	0.105	0.153		−0.006	0.937
SA	−0.016	0.833		−0.075	0.307
PN	0.057	0.437		0.026	0.720
EN	0.049	0.508		0.007	0.920
CTQ total score	0.083	0.262		−0.031	0.670
NPI	0.178	0.015*		−0.022	0.770
MI	0.232	0.001**		0.199	0.006**
CI	0.203	0.005**		0.053	0.474
BIS−11 total score	0.233	0.001**		0.068	0.358
AOE	−0.128	0.082		−0.085	0.247
ROE	−0.132	0.071		−0.225	0.018*
AE	0.040	0.589		−0.043	0.556
UE	−0.086	0.243		−0.106	0.150
EIS total score	−0.104	0.156		−0.099	0.180
PT	0.004	0.955		−0.026	0.722
PD	0.266	< 0.001***		0.225	0.002**
FS	−0.076	0.304		0.016	0.823
EC	−0.117	0.110		−0.001	0.984
IRI total score	0.022	0.766		0.060	0.416

*: *P* < 0.05; **: *P* < 0.01; ***: *P* < 0.001

**Table 4:** Pearson correlation among psychosocial variables

	1	2	3	4	5	6	7	8	9	10	11
NPI	-										
MI	0.407***	-									
CI	0.761**	0.508***	-								
AOE	−0.405***	−0.140	−0.414***	-							
AE	−0.364***	−0.128	−0.328***	0.512***	-						
PD	0.023	0.223**	0.072	−0.002	0.114	-					
PA	0.074	0.061	0.076	0.124	0.001	0.055	-				
EA	0.077	0.128	0.133	0.027	0.016	0.124	0.692***	-			
SA	0.116	−0.008	0.143	0.086	0.142	−0.036	0.331***	0.307***	-		
PN	0.158*	0.090	0.150*	−0.040	−0.098	−0.074	0.422***	0.526***	0.152*	-	
EN	0.164*	0.073	0.193**	−0.070	−0.139	−0.128	0.395***	0.599***	0.183*	0.668***	-
ROE	−0.459***	−0.108	−0.416***	0.566***	0.573***	0.019	−0.044	−0.015	0.005	−0.085	−0.088

*: *P* < 0.05; **: *P* < 0.01; ***: *P* < 0.001

### Mediation effect and structural equation model

Set MDD as an independent variable, NSSI as a dependent variable, and then set age and gender as covariates, and separately include motor impulsiveness and personal distress into the model for analysis. The results showed that total effect was significant (*p* = 0.012), motor impulsiveness (95% CI: 0.005,0.150) and personal distress (95% CI: 0.014,0.172) were partial intermediary variable that affects the relationship between MDD and NSSI (Table 5).

**Table 5:** Motor impulsiveness in the mediation effect analysis and the amount of effect

		Effect	Boot SE		T		*P*		95% CI			
									LLCI		ULCI	
Model 1 motor impulsiveness												
Total effect	0.338		0.133	2.535		0.012*		0.075		0.600		
Direct effect	0.276		0.133	2.068		0.040*		0.013		0.539		
Indirect effect	0.062		0.037	-		-		0.005		0.150		
Model 2 personal distress												
Total effect	0.338		0.133	2.535		0.012*		0.075		0.600		
Direct effect	0.262		0.133	1.974		0.050*		0.000		0.523		
Indirect effect	0.076		0.041	-		-		0.014		0.172		

*: *P* < 0.05

### Structural equation model results

In order to further understand how psychosocial factors affect the relationship between MDD and NSSI behavior, the structural equation model was constructed based on the above binary logical regression results, correlation analysis results and the known risk factors that affect the two main variables. The structural equation model assumes the following: (1) perspective taking and motor impulsiveness act as mediators between MDD and NSSI. (2) The social psychological factors with significant differences in the analysis of variance and the risk factors in the logistic regression interact with each other, like total score and each subscale of BIS-11, appraisal of own emotions, regulation of own emotions, appraisal of other’s emotions empathy, perspective taking, personal distress.

**Fig. 4:**
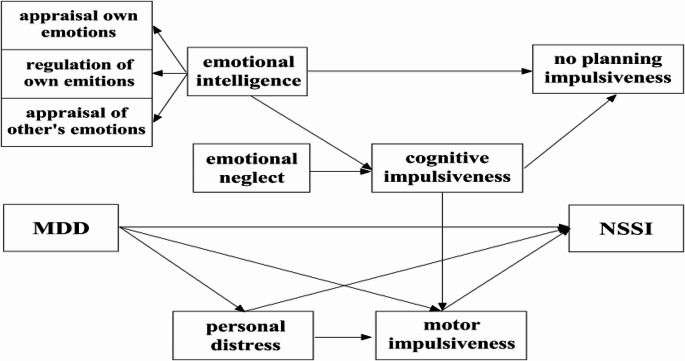
Structural equation model of the relationship between psychosocial factors and NSSI

The chi-square test of model fit value was 45.069, degrees of freedom = 40, P-value = 0.025, CMIN/DF = 1.127, RMSEA Estimate = 0.026, 90% confidence interval was 0.000 -0.059, CFI = 0.989, TLI = 0.986, IFI = 0.990, indicating a good fit. The results were shown in Fig. 4; Table 6. NSSI was directly affected by MDD severity (95%CI: 0.072,0.340), personal distress (95%CI: 0.064,0.346) and motor impulsiveness (95%CI: 0.013,0.300). At the same time, there were multiple effects between MDD severity and NSSI through personal distress (95%CI: 0.009,0.097) and motor impulsiveness (95%CI: 0.002,0.073). Emotional intelligence (95%CI: -0.124,-0.006), emotional neglect (95%CI: 0.000,0.006) and cognitive impulsiveness (95%CI: 0.006,0.162) had direct impacts on NSSI. Emotional intelligence (95%CI: -0.531,-0.124) directly affected no planning impulsiveness, and cognitive impulsiveness as an intermediary variable regulated the relationship between emotional intelligence and no planning impulsiveness (95%CI: -0.680,-0.286), as well as between emotional neglect and no planning impulsiveness (95%CI: 0.002,0.036).

**Table 6:** Relationships among multiple variables in the structural equation model

	Estimate	S.E.	*P*-Value	95% CI
HAMD→PD	0.196	0.067	0.006**	0.061,0.326
PD→MI	0.161	0.066	0.011*	0.035,0.288
HAMD→MI	0.136	0.067	0.025*	0.014,0.274
PD→NSSI	0.195	0.072	0.005**	0.064,0.346
MI→NSSI	0.152	0.072	0.034*	0.013,0.300
HAMD→NSSI	0.201	0.069	0.004**	0.072,0.340
EI→CI	-0.699	0.141	< 0.001***	-0.994,-0.450
EI→NPI	-0.303	0.102	< 0.001***	-0.531,-0.124
CI→MI	0.489	0.067	< 0.001***	0.354,0.619
CI→NPI	0.648	0.066	< 0.001***	0.508,0.763
EN→CI	0.028	0.012	0.023*	0.003,0.053
PN→CI	0.027	0.020	0.760	-0.146,0.236
PN→NPI	0.060	0.014	0.364	-0.074,0.197
EN→NPI	-0.023	0.009	0.689	-0.140,0.100
HAMD→PD→MI	0.032	0.016	0.007**	0.008,0.073
HAMD→PD→NSSI	0.040	0.022	0.010**	0.009,0.097
HAMD→CI→NSSI	0.025	0.017	0.029*	0.002,0.073
HAMD→PD→MI→NSSI	0.006	0.004	0.011*	0.001,0.022
EN→CI→MI	0.014	0.006	0.022*	0.002,0.027
EN→CI→NPI	0.018	0.008	0.021*	0.002,0.036
EN→CI→MI→NSSI	0.002	0.001	0.030*	0.000,0.006
EI→CI→MI	-0.342	0.080	< 0.001***	-0.515,-0.200
EI→CI→NPI	-0.453	0.099	< 0.001***	-0.680,-0.286
EI→CI→MI→NSSI	-0.052	0.029	0.027*	-0.124,-0.006
CI→MI→NSSI	0.074	0.038	0.031*	0.006,0.162
PD→MI→NSSI	0.025	0.016	0.018*	0.003,0.071
Gender→NSSI	0.195	0.061	0.004**	0.070,0.312

EI = Appraisal of own emotions + Regulation of own emotions + Appraisal of other’s emotions

*: *P* < 0.05; **: *P* < 0.01; ***: *P* < 0.001

## Discussion

This study is the first explore the multiple effects of childhood abuse, impulsiveness, emotional regulation and empathy on NSSI in MDD adolescents. More than half of adolescents with MDD had a history of NSSI and were younger than those without NSSI behavior, and their psychosocial factors were significantly different from those without NSSI behavior. MDD, personal distress, and motor impulsiveness had a direct impact on NSSI, but MDD had multiple mediating effects on NSSI behavior through personal distress and motor impulsiveness. Emotional intelligence, emotional neglect and cognitive impulsiveness had indirect impact on NSSI. It showed that childhood abuse, impulsiveness, emotional intelligence and empathy could predict the occurrence of NSSI behavior in MDD adolescents to a certain extent, so that we can have a deeper understanding of the psychosocial factors that affect the occurrence of NSSI behavior in MDD adolescents, thus providing a basis for the intervention of MDD adolescents with NSSI behavior.

In this study, the average age of adolescents with NSSI history was 15.99 years old, while adolescents without NSSI behavior were significantly older than those with. Previous study had shown that the prevalence of NSSI behavior reached its peak at the age of 15–16 years old and began to decline with age [29]. The study also found that 55.1% of adolescents had a history of NSSI, which was also consistent with the prevalence of NSSI among MDD adolescents in China.

Childhood abuse will have a series of adverse effects on children’s physical and mental health development, thus increasing the risk of risky behaviors among adolescents [30]. This study found that there was no significant difference in childhood trauma between MDD group and NSSI + group. The possible reason was that childhood trauma questionnaire requires subjects to recall their family experiences before the age of 16 [31]. At the same time, due to the differences in time background and epidemic situation and other factors, the subjects in this age group may be more coping with the pressure from academic, interpersonal and adaptive environment, which was regulated by other factors. In addition, they spend more time in the family than in school during the survey, teenagers’ experience of neglect and abuse was lower than that in the past. In the clinical sample, the above conditions may cause childhood abuse to have no significant effect between the severity of depression and NSSI behavior. A meta-analysis showed that the overall correlation between childhood trauma and NSSI was stronger among the subjects in the community sample. In addition, the study confirmed that emotional neglect could increase the risk of NSSI behavior in MDD adolescents indirectly [32, 33].

In the study, adolescents in the NSSI + group were significantly higher than those in the MDD group in all dimensions of impulsivity, and impulsivity was significantly correlated with NSSI and depression [34]. Among MDD adolescents, the higher the degree of motor impulsiveness, the teenagers tend to choose NSSI as the way of emotional regulation [35]. In addition, the study also found that cognitive impulsiveness was directly affected by emotional intelligence and emotional neglect, and cognitive impulsiveness promoted the generation of NSSI behavior by influencing motor impulsiveness. In the face of serious negative emotions, adolescents with high impulsiveness often choose to use NSSI to relieve their emotions, and this treatment mode is easy to make patients no longer consider other ways to deal with emotions, resulting in the weakening of the ability of emotion regulation strategies [36].

As a part of emotional intelligence, emotional regulation is closely related to NSSI [37, 38]. Adolescents with high emotional intelligence level will adjust and control their emotions when facing negative emotions or situations. However, adolescents with low emotional intelligence level have weak ability to control and regulate their emotions [39]. Adolescents with high emotional intelligence level use reasonable methods instead of NSSI to relieve their emotions. Adolescents with low emotional intelligence level implement NSSI behavior more frequently, thus relying on NSSI and Individuals cannot get rid of this simple and fast way, resulting in internal pain [40, 41]. The study also found that the relationship between emotional intelligence and NSSI was also mediated by impulsiveness. This indicates that MDD adolescents with NSSI need to learn and practice effective emotion and behavior regulation strategies in order to control individual impulse and achieve the goal of reducing NSSI [21].

Compared with MDD group, adolescents with NSSI showed less empathy and were more sensitive in personal distress dimension. Personal distress makes individuals feel anxiety and fear, and the purpose of individuals taking NSSI behavior is to reduce the impact of pain [42]. In the study, it was also found that personal distress played a mediating role between MDD and NSSI, that is, high level of personal distress may promote the occurrence of NSSI behavior in MDD adolescents. These findings indicate that personal distress, as a component of empathy, is also an important internal factor affecting the occurrence of NSSI behavior in MDD adolescents.

### Limitations

This study can provide evidence for clinical identification and intervention of MDD adolescents with NSSI behavior, but there are still some limitations. First of all, this study was a retrospective study, and there may be memory bias. Secondly, the functions and characteristics of NSSI may vary among different groups. This study recruited MDD adolescents with clinical samples, and should be careful when promoting the conclusion and applying it to social and school samples. Thirdly, because this experiment is a cross-sectional study, it is possible to draw a precise conclusion about the causal relationship between MDD and between various psychosocial factors. Future research can increase intervention and follow-up to explore the causal relationship between psychosocial factors and MDD and NSSI. Finally, future research needs a larger sample size to verify the results.

## Conclusion

A total of 55.1% of MDD adolescents had a history of NSSI. MDD adolescents with a history of NSSI have many differences in impulse, empathy and emotional intelligence compared with MDD adolescents without NSSI behavior. Impulsiveness, personal distress, emotional neglect, and emotional intelligence are important social and psychological factors that affect NSSI behavior in adolescents with MDD. These differences affect the occurrence of NSSI through chain mediation. In the future, active attention should be paid to the above risk factors in this population, so as to achieve early identification and intervention for such adolescents, in order to effectively improve their social functions.

## Data Availability

The data generated in this study are available from the corresponding author upon reasonable request.
